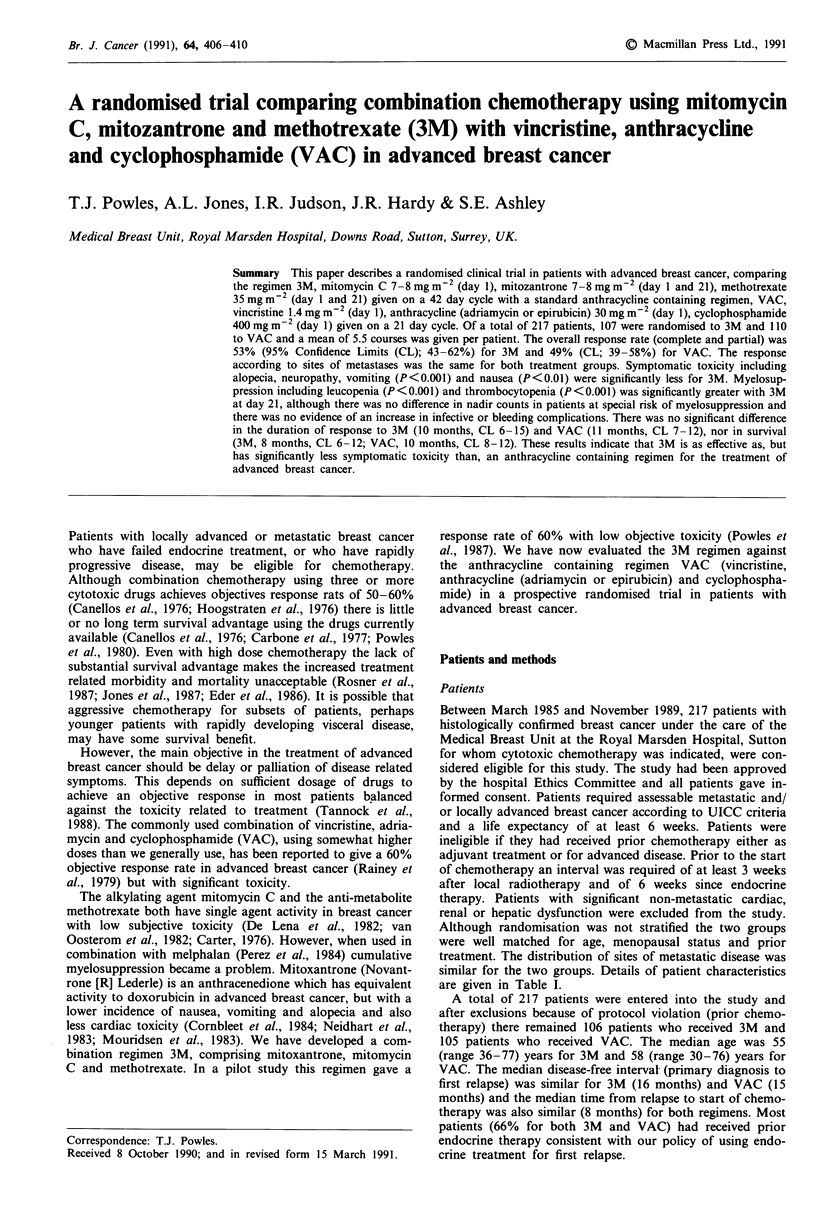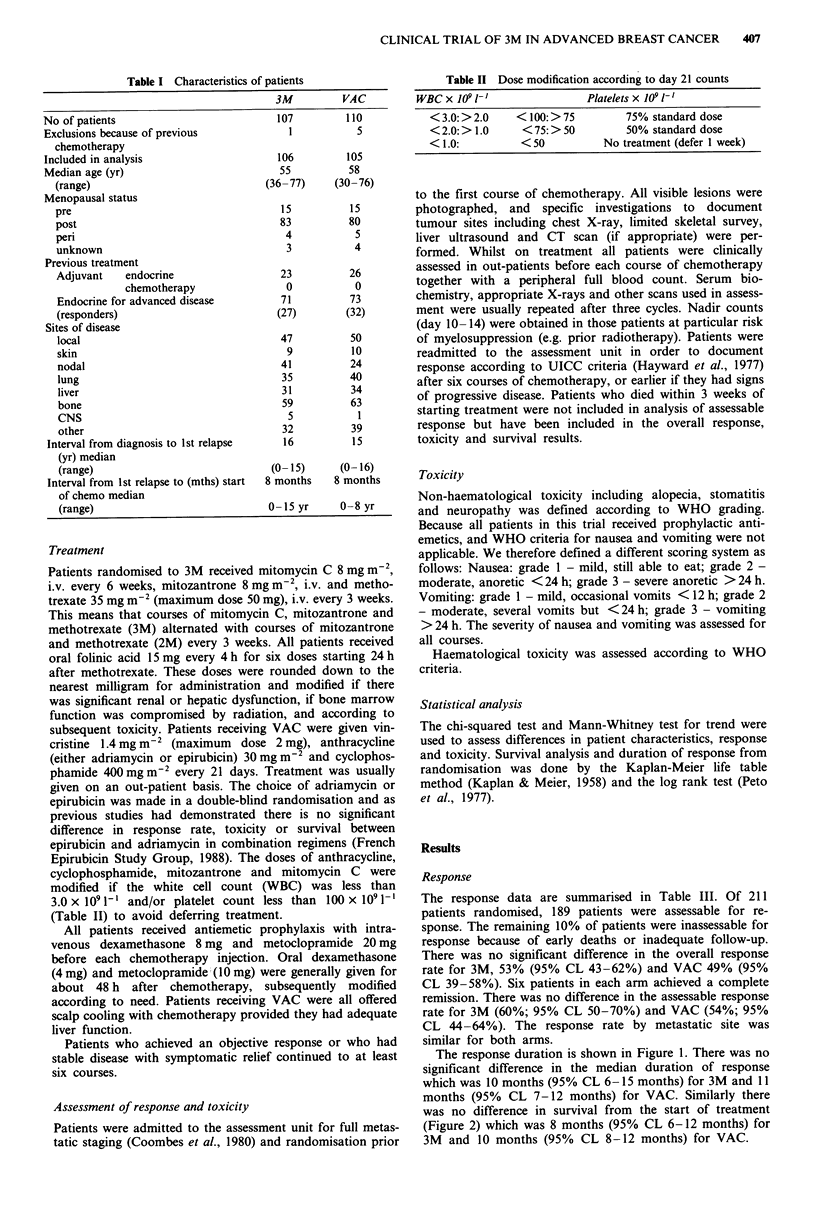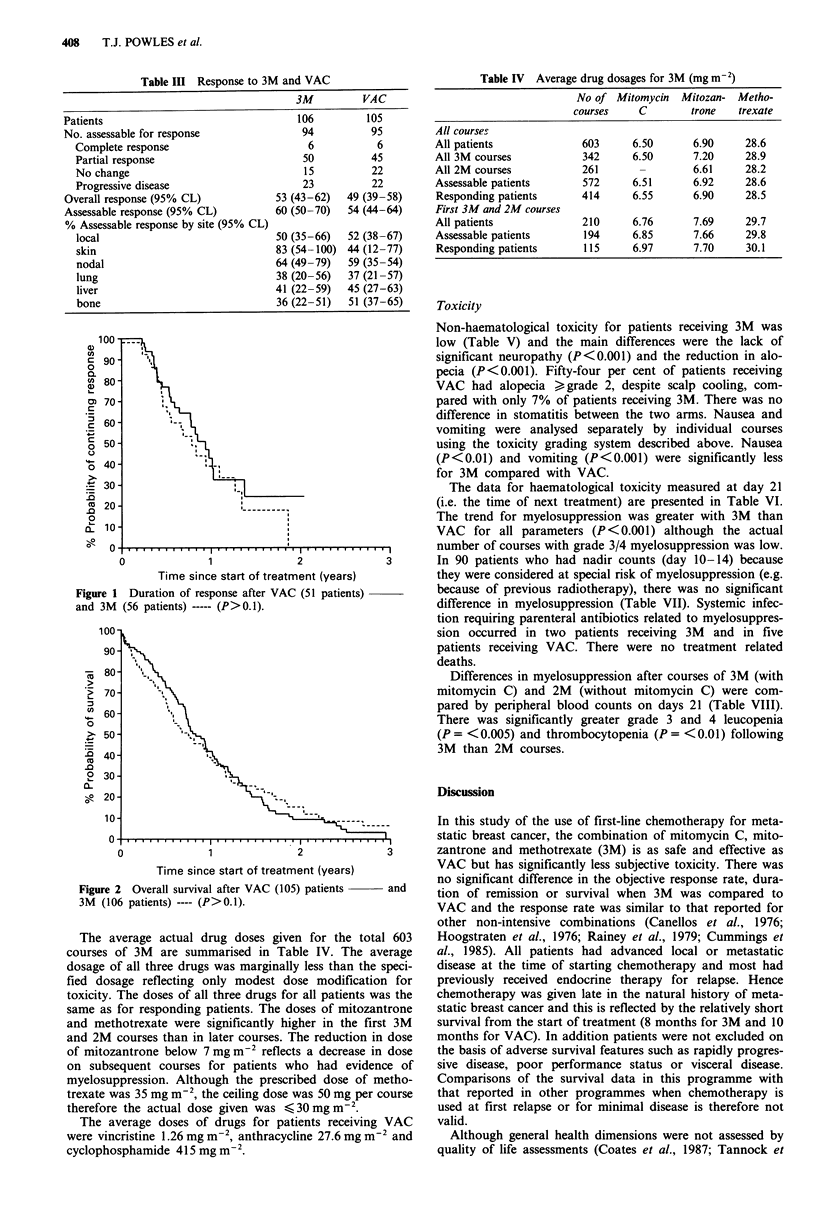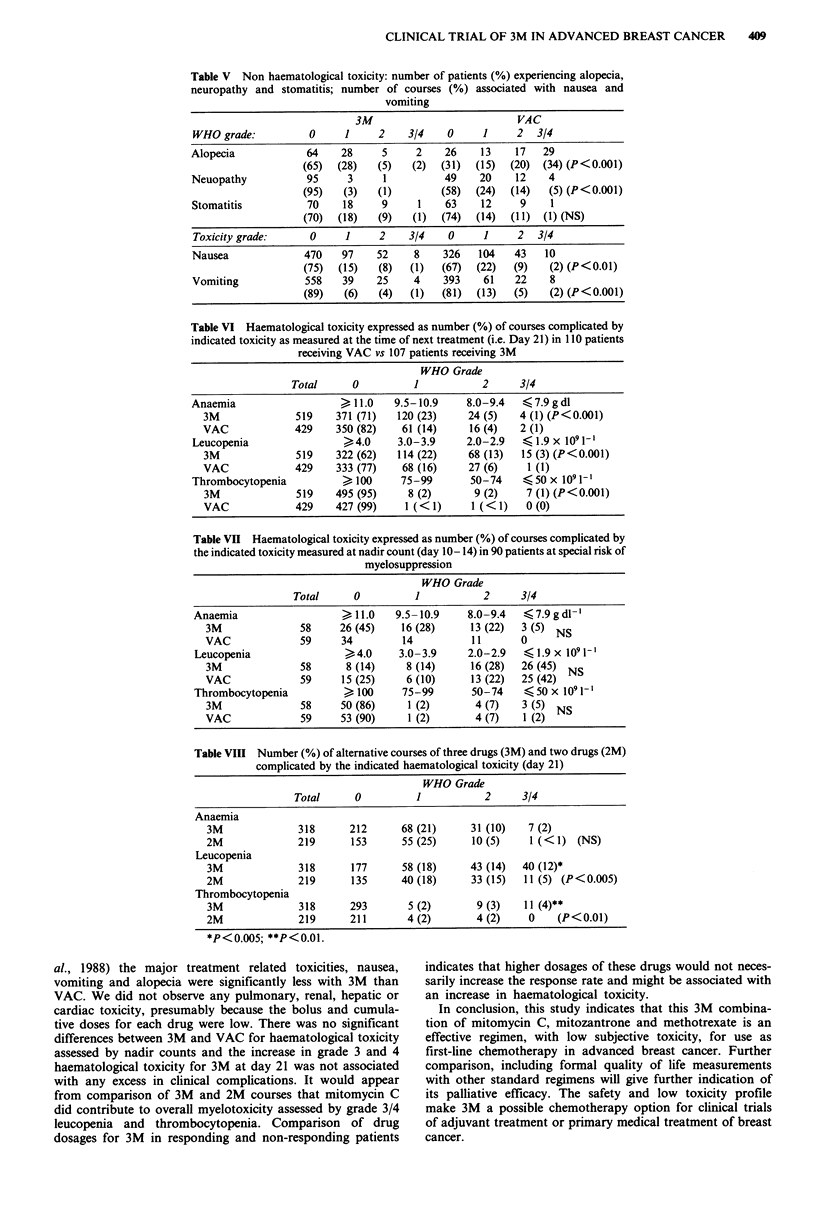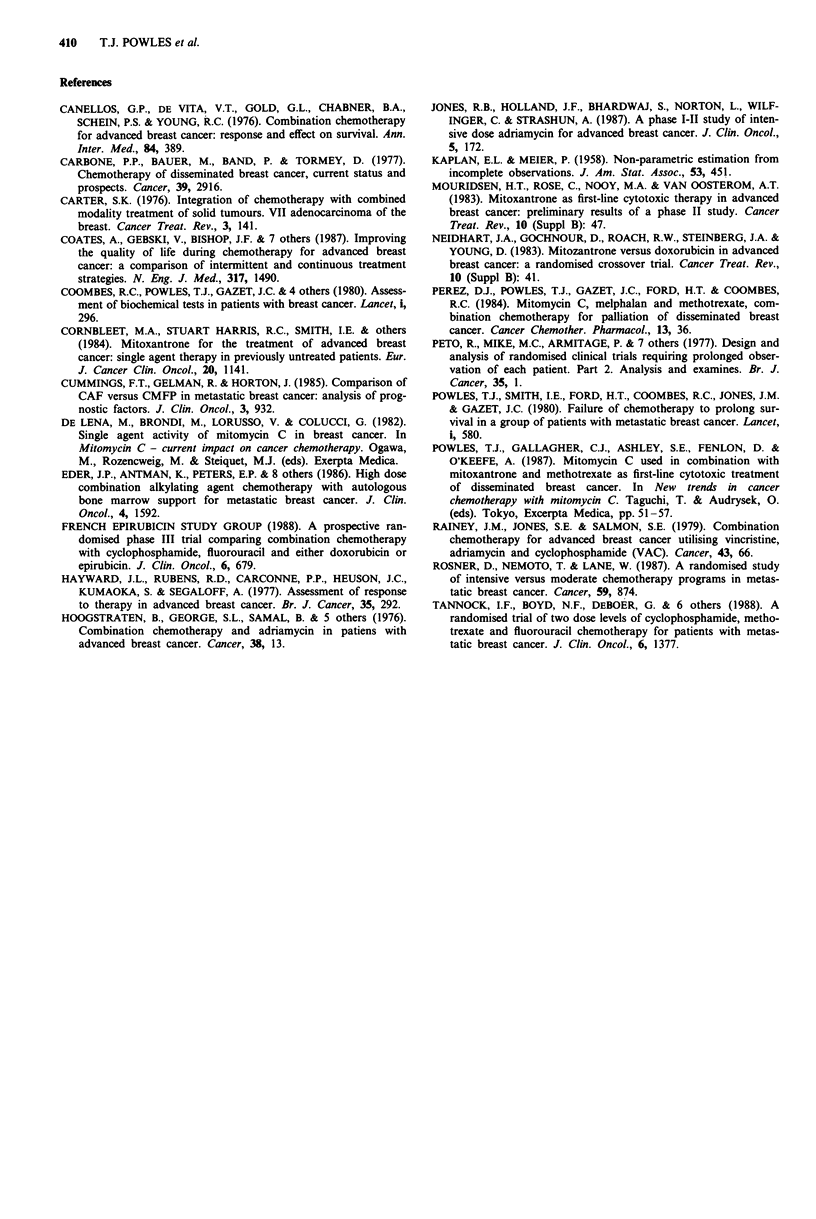# A randomised trial comparing combination chemotherapy using mitomycin C, mitozantrone and methotrexate (3M) with vincristine, anthracycline and cyclophosphamide (VAC) in advanced breast cancer.

**DOI:** 10.1038/bjc.1991.318

**Published:** 1991-08

**Authors:** T. J. Powles, A. L. Jones, I. R. Judson, J. R. Hardy, S. E. Ashley

**Affiliations:** Medical Breast Unit, Royal Marsden Hospital, Sutton, Surrey, UK.

## Abstract

This paper describes a randomised clinical trial in patients with advanced breast cancer, comparing the regimen 3M, mitomycin C 7-8 mg m-2 (day 1), mitozantrone 7-8 mg m-2 (day 1 and 21), methotrexate 35 mg m-2 (day 1 and 21) given on a 42 day cycle with a standard anthracycline containing regimen, VAC, vincristine 1.4 mg m-2 (day 1), anthracycline (adriamycin or epirubicin) 30 mg m-2 (day 1), cyclophosphamide 400 mg m-2 (day 1) given on a 21 day cycle. Of a total of 217 patients, 107 were randomised to 3M and 110 to VAC and a mean of 5.5 courses was given per patient. The overall response rate (complete and partial) was 53% (95% Confidence Limits (CL): 43-62%) for 3M and 49% (CL; 39-58%) for VAC. The response according to sites of metastases was the same for both treatment groups. Symptomatic toxicity including alopecia, neuropathy, vomiting (P less than 0.001) and nausea (P less than 0.01) were significantly less for 3M. Myelosuppression including leucopenia (P less than 0.001) and thrombocytopenia (P less than 0.001) was significantly greater with 3M at day 21, although there was no difference in nadir counts in patients at special risk of myelosuppression and there was no evidence of an increase in infective or bleeding complications. There was no significant difference in the duration of response to 3M (10 months, CL 6-15) and VAC (11 months, CL 7-12), nor in survival (3M, 8 months, CL 6-12; VAC, 10 months, CL 8-12). These results indicate that 3M is as effective as, but has significantly less symptomatic toxicity than, an anthracycline containing regimen for the treatment of advanced breast cancer.


Br. J. Cancer (1991), 64, 406-410 © Macmillan Press Ltd., 1991

### A randomised trial comparing combination chemotherapy using mitomycin C, mitozantrone and methotrexate (3M) with vincristine, anthracycline and cyclophosphamide (VAC) in advanced breast cancer

T.J. Powles, A.L. Jones, I.R. Judson, J.R. Hardy & S.E. Ashley

*Medical Breast Unit, Royal Marsden Hospital, Downs Road, Sutton, Surrey, UK.*

**Summary** This paper describes a randomised clinical trial in patients with advanced breast cancer, comparing the regimen 3M, mitomycin C 7–8 mg $m^{-2}$ (day 1), mitozantrone 7–8 mg $m^{-2}$ (day 1 and 21), methotrexate 35 mg $m^{-2}$ (day 1 and 21) given on a 42 day cycle with a standard anthracycline containing regimen, VAC, vincristine 1.4 mg $m^{-2}$ (day 1), anthracycline (adriamycin or epirubicin) 30 mg $m^{-2}$ (day 1), cyclophosphamide 400 mg $m^{-2}$ (day 1) given on a 21 day cycle. Of a total of 217 patients, 107 were randomised to 3M and 110 to VAC and a mean of 5.5 courses was given per patient. The overall response rate (complete and partial) was 53% (95% Confidence Limits (CL); 43–62%) for 3M and 49% (CL; 39–58%) for VAC. The response according to sites of metastases was the same for both treatment groups. Symptomatic toxicity including alopecia, neuropathy, vomiting ($P<0.001$) and nausea ($P<0.01$) were significantly less for 3M. Myelosuppression including leucopenia ($P<0.001$) and thrombocytopenia ($P<0.001$) was significantly greater with 3M at day 21, although there was no difference in nadir counts in patients at special risk of myelosuppression and there was no evidence of an increase in infective or bleeding complications. There was no significant difference in the duration of response to 3M (10 months, CL 6–15) and VAC (11 months, CL 7–12), nor in survival (3M, 8 months, CL 6–12; VAC, 10 months, CL 8–12). These results indicate that 3M is as effective as, but has significantly less symptomatic toxicity than, an anthracycline containing regimen for the treatment of advanced breast cancer.

Patients with locally advanced or metastatic breast cancer who have failed endocrine treatment, or who have rapidly progressive disease, may be eligible for chemotherapy. Although combination chemotherapy using three or more cytotoxic drugs achieves objectives response rats of 50–60% (Canellos *et al.*, 1976; Hoogstraten *et al.*, 1976) there is little or no long term survival advantage using the drugs currently available (Canellos *et al.*, 1976; Carbone *et al.*, 1977; Powles *et al.*, 1980). Even with high dose chemotherapy the lack of substantial survival advantage makes the increased treatment related morbidity and mortality unacceptable (Rosner *et al.*, 1987; Jones *et al.*, 1987; Eder *et al.*, 1986). It is possible that aggressive chemotherapy for subsets of patients, perhaps younger patients with rapidly developing visceral disease, may have some survival benefit.

However, the main objective in the treatment of advanced breast cancer should be delay or palliation of disease related symptoms. This depends on sufficient dosage of drugs to achieve an objective response in most patients balanced against the toxicity related to treatment (Tannock *et al.*, 1988). The commonly used combination of vincristine, adriamycin and cyclophosphamide (VAC), using somewhat higher doses than we generally use, has been reported to give a 60% objective response rate in advanced breast cancer (Rainey *et al.*, 1979) but with significant toxicity.

The alkylating agent mitomycin C and the anti-metabolite methotrexate both have single agent activity in breast cancer with low subjective toxicity (De Lena *et al.*, 1982; van Oosterom *et al.*, 1982; Carter, 1976). However, when used in combination with melphalan (Perez *et al.*, 1984) cumulative myelosuppression became a problem. Mitoxantrone (Novantrone [R] Lederle) is an anthracenedione which has equivalent activity to doxorubicin in advanced breast cancer, but with a lower incidence of nausea, vomiting and alopecia and also less cardiac toxicity (Cornbleet *et al.*, 1984; Neidhart *et al.*, 1983; Mouridsen *et al.*, 1983). We have developed a combination regimen 3M, comprising mitoxantrone, mitomycin C and methotrexate. In a pilot study this regimen gave a response rate of 60% with low objective toxicity (Powles *et al.*, 1987). We have now evaluated the 3M regimen against the anthracycline containing regimen VAC (vincristine, anthracycline (adriamycin or epirubicin) and cyclophosphamide) in a prospective randomised trial in patients with advanced breast cancer.

Correspondence: T.J. Powles.
Received 8 October 1990; and in revised form 15 March 1991.

### Patients and methods

### *Patients*

Between March 1985 and November 1989, 217 patients with histologically confirmed breast cancer under the care of the Medical Breast Unit at the Royal Marsden Hospital, Sutton for whom cytotoxic chemotherapy was indicated, were considered eligible for this study. The study had been approved by the hospital Ethics Committee and all patients gave informed consent. Patients required assessable metastatic and/or locally advanced breast cancer according to UICC criteria and a life expectancy of at least 6 weeks. Patients were ineligible if they had received prior chemotherapy either as adjuvant treatment or for advanced disease. Prior to the start of chemotherapy an interval was required of at least 3 weeks after local radiotherapy and of 6 weeks since endocrine therapy. Patients with significant non-metastatic cardiac, renal or hepatic dysfunction were excluded from the study. Although randomisation was not stratified the two groups were well matched for age, menopausal status and prior treatment. The distribution of sites of metastatic disease was similar for the two groups. Details of patient characteristics are given in Table I.

A total of 217 patients were entered into the study and after exclusions because of protocol violation (prior chemotherapy) there remained 106 patients who received 3M and 105 patients who received VAC. The median age was 55 (range 36–77) years for 3M and 58 (range 30–76) years for VAC. The median disease-free interval (primary diagnosis to first relapse) was similar for 3M (16 months) and VAC (15 months) and the median time from relapse to start of chemotherapy was also similar (8 months) for both regimens. Most patients (66% for both 3M and VAC) had received prior endocrine therapy consistent with our policy of using endocrine treatment for first relapse.

**Table I** Characteristics of patients

| | *3M* | *VAC* |
|---|---|---|
| No of patients | 107 | 110 |
| Exclusions because of previous chemotherapy | 1 | 5 |
| Included in analysis | 106 | 105 |
| Median age (yr) | 55 | 58 |
| (range) | (36–77) | (30–76) |
| Menopausal status | | |
| pre | 15 | 15 |
| post | 83 | 80 |
| peri | 4 | 5 |
| unknown | 3 | 4 |
| Previous treatment | | |
| Adjuvant endocrine | 23 | 26 |
| chemotherapy | 0 | 0 |
| Endocrine for advanced disease | 71 | 73 |
| (responders) | (27) | (32) |
| Sites of disease | | |
| local | 47 | 50 |
| skin | 9 | 10 |
| nodal | 41 | 24 |
| lung | 35 | 40 |
| liver | 31 | 34 |
| bone | 59 | 63 |
| CNS | 5 | 1 |
| other | 32 | 39 |
| Interval from diagnosis to 1st relapse (yr) median | 16 | 15 |
| (range) | (0–15) | (0–16) |
| Interval from 1st relapse to (mths) start of chemo median | 8 months | 8 months |
| (range) | 0–15 yr | 0–8 yr |

*Treatment*

Patients randomised to 3M received mitomycin C 8 mg m$^{-2}$, i.v. every 6 weeks, mitozantrone 8 mg m$^{-2}$, i.v. and methotrexate 35 mg m$^{-2}$ (maximum dose 50 mg), i.v. every 3 weeks. This means that courses of mitomycin C, mitozantrone and methotrexate (3M) alternated with courses of mitozantrone and methotrexate (2M) every 3 weeks. All patients received oral folinic acid 15 mg every 4 h for six doses starting 24 h after methotrexate. These doses were rounded down to the nearest milligram for administration and modified if there was significant renal or hepatic dysfunction, if bone marrow function was compromised by radiation, and according to subsequent toxicity. Patients receiving VAC were given vincristine 1.4 mg m$^{-2}$ (maximum dose 2 mg), anthracycline (either adriamycin or epirubicin) 30 mg m$^{-2}$ and cyclophosphamide 400 mg m$^{-2}$ every 21 days. Treatment was usually given on an out-patient basis. The choice of adriamycin or epirubicin was made in a double-blind randomisation and as previous studies had demonstrated there is no significant difference in response rate, toxicity or survival between epirubicin and adriamycin in combination regimens (French Epirubicin Study Group, 1988). The doses of anthracycline, cyclophosphamide, mitozantrone and mitomycin C were modified if the white cell count (WBC) was less than $3.0 \times 10^9\,l^{-1}$ and/or platelet count less than $100 \times 10^9\,l^{-1}$ (Table II) to avoid deferring treatment.

All patients received antiemetic prophylaxis with intravenous dexamethasone 8 mg and metoclopramide 20 mg before each chemotherapy injection. Oral dexamethasone (4 mg) and metoclopramide (10 mg) were generally given for about 48 h after chemotherapy, subsequently modified according to need. Patients receiving VAC were all offered scalp cooling with chemotherapy provided they had adequate liver function.

Patients who achieved an objective response or who had stable disease with symptomatic relief continued to at least six courses.

*Assessment of response and toxicity*

Patients were admitted to the assessment unit for full metastatic staging (Coombes *et al.*, 1980) and randomisation prior to the first course of chemotherapy. All visible lesions were photographed, and specific investigations to document tumour sites including chest X-ray, limited skeletal survey, liver ultrasound and CT scan (if appropriate) were performed. Whilst on treatment all patients were clinically assessed in out-patients before each course of chemotherapy together with a peripheral full blood count. Serum biochemistry, appropriate X-rays and other scans used in assessment were usually repeated after three cycles. Nadir counts (day 10–14) were obtained in those patients at particular risk of myelosuppression (e.g. prior radiotherapy). Patients were readmitted to the assessment unit in order to document response according to UICC criteria (Hayward *et al.*, 1977) after six courses of chemotherapy, or earlier if they had signs of progressive disease. Patients who died within 3 weeks of starting treatment were not included in analysis of assessable response but have been included in the overall response, toxicity and survival results.

**Table II** Dose modification according to day 21 counts

| *WBC × $10^9\,l^{-1}$* | *Platelets × $10^9\,l^{-1}$* | |
|---|---|---|
| <3.0:>2.0 | <100:>75 | 75% standard dose |
| <2.0:>1.0 | <75:>50 | 50% standard dose |
| <1.0: | <50 | No treatment (defer 1 week) |

*Toxicity*

Non-haematological toxicity including alopecia, stomatitis and neuropathy was defined according to WHO grading. Because all patients in this trial received prophylactic anti-emetics, and WHO criteria for nausea and vomiting were not applicable. We therefore defined a different scoring system as follows: Nausea: grade 1 – mild, still able to eat; grade 2 – moderate, anoretic <24 h; grade 3 – severe anoretic >24 h. Vomiting: grade 1 – mild, occasional vomits <12 h; grade 2 – moderate, several vomits but <24 h; grade 3 – vomiting >24 h. The severity of nausea and vomiting was assessed for all courses.

Haematological toxicity was assessed according to WHO criteria.

*Statistical analysis*

The chi-squared test and Mann-Whitney test for trend were used to assess differences in patient characteristics, response and toxicity. Survival analysis and duration of response from randomisation was done by the Kaplan-Meier life table method (Kaplan & Meier, 1958) and the log rank test (Peto *et al.*, 1977).

### Results

*Response*

The response data are summarised in Table III. Of 211 patients randomised, 189 patients were assessable for response. The remaining 10% of patients were inassessable for response because of early deaths or inadequate follow-up. There was no significant difference in the overall response rate for 3M, 53% (95% CL 43–62%) and VAC 49% (95% CL 39–58%). Six patients in each arm achieved a complete remission. There was no difference in the assessable response rate for 3M (60%; 95% CL 50–70%) and VAC (54%; 95% CL 44–64%). The response rate by metastatic site was similar for both arms.

The response duration is shown in Figure 1. There was no significant difference in the median duration of response which was 10 months (95% CL 6–15 months) for 3M and 11 months (95% CL 7–12 months) for VAC. Similarly there was no difference in survival from the start of treatment (Figure 2) which was 8 months (95% CL 6–12 months) for 3M and 10 months (95% CL 8–12 months) for VAC.

**Table III** Response to 3M and VAC

| | *3M* | *VAC* |
|---|---|---|
| Patients | 106 | 105 |
| No. assessable for response | 94 | 95 |
| Complete response | 6 | 6 |
| Partial response | 50 | 45 |
| No change | 15 | 22 |
| Progressive disease | 23 | 22 |
| Overall response (95% CL) | 53 (43-62) | 49 (39-58) |
| Assessable response (95% CL) | 60 (50-70) | 54 (44-64) |
| % Assessable response by site (95% CL) | | |
| local | 50 (35-66) | 52 (38-67) |
| skin | 83 (54-100) | 44 (12-77) |
| nodal | 64 (49-79) | 59 (35-54) |
| lung | 38 (20-56) | 37 (21-57) |
| liver | 41 (22-59) | 45 (27-63) |
| bone | 36 (22-51) | 51 (37-65) |

**Figure 1** Duration of response after VAC (51 patients) ——— and 3M (56 patients) ----- ($P > 0.1$).

**Figure 2** Overall survival after VAC (105) patients ——— and 3M (106 patients) ---- ($P > 0.1$).

The average actual drug doses given for the total 603 courses of 3M are summarised in Table IV. The average dosage of all three drugs was marginally less than the specified dosage reflecting only modest dose modification for toxicity. The doses of all three drugs for all patients was the same as for responding patients. The doses of mitozantrone and methotrexate were significantly higher in the first 3M and 2M courses than in later courses. The reduction in dose of mitozantrone below 7 mg $m^{-2}$ reflects a decrease in dose on subsequent courses for patients who had evidence of myelosuppression. Although the prescribed dose of methotrexate was 35 mg $m^{-2}$, the ceiling dose was 50 mg per course therefore the actual dose given was $\leqslant 30$ mg $m^{-2}$.

The average doses of drugs for patients receiving VAC were vincristine 1.26 mg $m^{-2}$, anthracycline 27.6 mg $m^{-2}$ and cyclophosphamide 415 mg $m^{-2}$.

**Table IV** Average drug dosages for 3M (mg $m^{-2}$)

| | *No of courses* | *Mitomycin C* | *Mitozantrone* | *Methotrexate* |
|---|---|---|---|---|
| *All courses* | | | | |
| All patients | 603 | 6.50 | 6.90 | 28.6 |
| All 3M courses | 342 | 6.50 | 7.20 | 28.9 |
| All 2M courses | 261 | – | 6.61 | 28.2 |
| Assessable patients | 572 | 6.51 | 6.92 | 28.6 |
| Responding patients | 414 | 6.55 | 6.90 | 28.5 |
| *First 3M and 2M courses* | | | | |
| All patients | 210 | 6.76 | 7.69 | 29.7 |
| Assessable patients | 194 | 6.85 | 7.66 | 29.8 |
| Responding patients | 115 | 6.97 | 7.70 | 30.1 |

### *Toxicity*

Non-haematological toxicity for patients receiving 3M was low (Table V) and the main differences were the lack of significant neuropathy ($P < 0.001$) and the reduction in alopecia ($P < 0.001$). Fifty-four per cent of patients receiving VAC had alopecia $\geqslant$grade 2, despite scalp cooling, compared with only 7% of patients receiving 3M. There was no difference in stomatitis between the two arms. Nausea and vomiting were analysed separately by individual courses using the toxicity grading system described above. Nausea ($P < 0.01$) and vomiting ($P < 0.001$) were significantly less for 3M compared with VAC.

The data for haematological toxicity measured at day 21 (i.e. the time of next treatment) are presented in Table VI. The trend for myelosuppression was greater with 3M than VAC for all parameters ($P < 0.001$) although the actual number of courses with grade 3/4 myelosuppression was low. In 90 patients who had nadir counts (day 10–14) because they were considered at special risk of myelosuppression (e.g. because of previous radiotherapy), there was no significant difference in myelosuppression (Table VII). Systemic infection requiring parenteral antibiotics related to myelosuppression occurred in two patients receiving 3M and in five patients receiving VAC. There were no treatment related deaths.

Differences in myelosuppression after courses of 3M (with mitomycin C) and 2M (without mitomycin C) were compared by peripheral blood counts on days 21 (Table VIII). There was significantly greater grade 3 and 4 leucopenia ($P = < 0.005$) and thrombocytopenia ($P = < 0.01$) following 3M than 2M courses.

### Discussion

In this study of the use of first-line chemotherapy for metastatic breast cancer, the combination of mitomycin C, mitozantrone and methotrexate (3M) is as safe and effective as VAC but has significantly less subjective toxicity. There was no significant difference in the objective response rate, duration of remission or survival when 3M was compared to VAC and the response rate was similar to that reported for other non-intensive combinations (Canellos *et al.*, 1976; Hoogstraten *et al.*, 1976; Rainey *et al.*, 1979; Cummings *et al.*, 1985). All patients had advanced local or metastatic disease at the time of starting chemotherapy and most had previously received endocrine therapy for relapse. Hence chemotherapy was given late in the natural history of metastatic breast cancer and this is reflected by the relatively short survival from the start of treatment (8 months for 3M and 10 months for VAC). In addition patients were not excluded on the basis of adverse survival features such as rapidly progressive disease, poor performance status or visceral disease. Comparisons of the survival data in this programme with that reported in other programmes when chemotherapy is used at first relapse or for minimal disease is therefore not valid.

Although general health dimensions were not assessed by quality of life assessments (Coates *et al.*, 1987; Tannock *et*

**Table V** Non haematological toxicity: number of patients (%) experiencing alopecia, neuropathy and stomatitis; number of courses (%) associated with nausea and vomiting

| | 3M | | | | VAC | | | |
|---|---|---|---|---|---|---|---|---|
| *WHO grade:* | 0 | 1 | 2 | 3/4 | 0 | 1 | 2 | 3/4 |
| Alopecia | 64 | 28 | 5 | 2 | 26 | 13 | 17 | 29 |
| | (65) | (28) | (5) | (2) | (31) | (15) | (20) | (34) ($P<0.001$) |
| Neuopathy | 95 | 3 | 1 | | 49 | 20 | 12 | 4 |
| | (95) | (3) | (1) | | (58) | (24) | (14) | (5) ($P<0.001$) |
| Stomatitis | 70 | 18 | 9 | 1 | 63 | 12 | 9 | 1 |
| | (70) | (18) | (9) | (1) | (74) | (14) | (11) | (1) (NS) |
| *Toxicity grade:* | 0 | 1 | 2 | 3/4 | 0 | 1 | 2 | 3/4 |
| Nausea | 470 | 97 | 52 | 8 | 326 | 104 | 43 | 10 |
| | (75) | (15) | (8) | (1) | (67) | (22) | (9) | (2) ($P<0.01$) |
| Vomiting | 558 | 39 | 25 | 4 | 393 | 61 | 22 | 8 |
| | (89) | (6) | (4) | (1) | (81) | (13) | (5) | (2) ($P<0.001$) |

**Table VI** Haematological toxicity expressed as number (%) of courses complicated by indicated toxicity as measured at the time of next treatment (i.e. Day 21) in 110 patients receiving VAC *vs* 107 patients receiving 3M

| | | WHO Grade | | | |
|---|---|---|---|---|---|
| | *Total* | *0* | *1* | *2* | *3/4* |
| Anaemia | | ⩾11.0 | 9.5–10.9 | 8.0–9.4 | ⩽7.9 g dl |
| 3M | 519 | 371 (71) | 120 (23) | 24 (5) | 4 (1) ($P<0.001$) |
| VAC | 429 | 350 (82) | 61 (14) | 16 (4) | 2 (1) |
| Leucopenia | | ⩾4.0 | 3.0–3.9 | 2.0–2.9 | ⩽$1.9\times10^9 l^{-1}$ |
| 3M | 519 | 322 (62) | 114 (22) | 68 (13) | 15 (3) ($P<0.001$) |
| VAC | 429 | 333 (77) | 68 (16) | 27 (6) | 1 (1) |
| Thrombocytopenia | | ⩾100 | 75–99 | 50–74 | ⩽$50\times10^9 l^{-1}$ |
| 3M | 519 | 495 (95) | 8 (2) | 9 (2) | 7 (1) ($P<0.001$) |
| VAC | 429 | 427 (99) | 1 (<1) | 1 (<1) | 0 (0) |

**Table VII** Haematological toxicity expressed as number (%) of courses complicated by the indicated toxicity measured at nadir count (day 10–14) in 90 patients at special risk of myelosuppression

| | | WHO Grade | | | |
|---|---|---|---|---|---|
| | *Total* | *0* | *1* | *2* | *3/4* |
| Anaemia | | ⩾11.0 | 9.5–10.9 | 8.0–9.4 | ⩽7.9 g $dl^{-1}$ |
| 3M | 58 | 26 (45) | 16 (28) | 13 (22) | 3 (5) NS |
| VAC | 59 | 34 | 14 | 11 | 0 |
| Leucopenia | | ⩾4.0 | 3.0–3.9 | 2.0–2.9 | ⩽$1.9\times10^9 l^{-1}$ |
| 3M | 58 | 8 (14) | 8 (14) | 16 (28) | 26 (45) NS |
| VAC | 59 | 15 (25) | 6 (10) | 13 (22) | 25 (42) |
| Thrombocytopenia | | ⩾100 | 75–99 | 50–74 | ⩽$50\times10^9 l^{-1}$ |
| 3M | 58 | 50 (86) | 1 (2) | 4 (7) | 3 (5) NS |
| VAC | 59 | 53 (90) | 1 (2) | 4 (7) | 1 (2) |

**Table VIII** Number (%) of alternative courses of three drugs (3M) and two drugs (2M) complicated by the indicated haematological toxicity (day 21)

| | | WHO Grade | | | |
|---|---|---|---|---|---|
| | *Total* | *0* | *1* | *2* | *3/4* |
| Anaemia | | | | | |
| 3M | 318 | 212 | 68 (21) | 31 (10) | 7 (2) |
| 2M | 219 | 153 | 55 (25) | 10 (5) | 1 (<1) (NS) |
| Leucopenia | | | | | |
| 3M | 318 | 177 | 58 (18) | 43 (14) | 40 (12)* |
| 2M | 219 | 135 | 40 (18) | 33 (15) | 11 (5) ($P<0.005$) |
| Thrombocytopenia | | | | | |
| 3M | 318 | 293 | 5 (2) | 9 (3) | 11 (4)** |
| 2M | 219 | 211 | 4 (2) | 4 (2) | 0 ($P<0.01$) |

*$P<0.005$; **$P<0.01$.

*al.*, 1988) the major treatment related toxicities, nausea, vomiting and alopecia were significantly less with 3M than VAC. We did not observe any pulmonary, renal, hepatic or cardiac toxicity, presumably because the bolus and cumulative doses for each drug were low. There was no significant differences between 3M and VAC for haematological toxicity assessed by nadir counts and the increase in grade 3 and 4 haematological toxicity for 3M at day 21 was not associated with any excess in clinical complications. It would appear from comparison of 3M and 2M courses that mitomycin C did contribute to overall myelotoxicity assessed by grade 3/4 leucopenia and thrombocytopenia. Comparison of drug dosages for 3M in responding and non-responding patients indicates that higher dosages of these drugs would not necessarily increase the response rate and might be associated with an increase in haematological toxicity.

In conclusion, this study indicates that this 3M combination of mitomycin C, mitozantrone and methotrexate is an effective regimen, with low subjective toxicity, for use as first-line chemotherapy in advanced breast cancer. Further comparison, including formal quality of life measurements with other standard regimens will give further indication of its palliative efficacy. The safety and low toxicity profile make 3M a possible chemotherapy option for clinical trials of adjuvant treatment or primary medical treatment of breast cancer.

### References

CANELLOS, G.P., DE VITA, V.T., GOLD, G.L., CHABNER, B.A., SCHEIN, P.S. & YOUNG, R.C. (1976). Combination chemotherapy for advanced breast cancer: response and effect on survival. *Ann. Inter. Med.*, **84**, 389.

CARBONE, P.P., BAUER, M., BAND, P. & TORMEY, D. (1977). Chemotherapy of disseminated breast cancer, current status and prospects. *Cancer*, **39**, 2916.

CARTER, S.K. (1976). Integration of chemotherapy with combined modality treatment of solid tumours. VII adenocarcinoma of the breast. *Cancer Treat. Rev.*, **3**, 141.

COATES, A., GEBSKI, V., BISHOP, J.F. & 7 others (1987). Improving the quality of life during chemotherapy for advanced breast cancer: a comparison of intermittent and continuous treatment strategies. *N. Eng. J. Med.*, **317**, 1490.

COOMBES, R.C., POWLES, T.J., GAZET, J.C. & 4 others (1980). Assessment of biochemical tests in patients with breast cancer. *Lancet*, **i**, 296.

CORNBLEET, M.A., STUART HARRIS, R.C., SMITH, I.E. & others (1984). Mitoxantrone for the treatment of advanced breast cancer: single agent therapy in previously untreated patients. *Eur. J. Cancer Clin. Oncol.*, **20**, 1141.

CUMMINGS, F.T., GELMAN, R. & HORTON, J. (1985). Comparison of CAF versus CMFP in metastatic breast cancer: analysis of prognostic factors. *J. Clin. Oncol.*, **3**, 932.

DE LENA, M., BRONDI, M., LORUSSO, V. & COLUCCI, G. (1982). Single agent activity of mitomycin C in breast cancer. In *Mitomycin C – current impact on cancer chemotherapy*. Ogawa, M., Rozencweig, M. & Steiquet, M.J. (eds). Exerpta Medica.

EDER, J.P., ANTMAN, K., PETERS, E.P. & 8 others (1986). High dose combination alkylating agent chemotherapy with autologous bone marrow support for metastatic breast cancer. *J. Clin. Oncol.*, **4**, 1592.

FRENCH EPIRUBICIN STUDY GROUP (1988). A prospective randomised phase III trial comparing combination chemotherapy with cyclophosphamide, fluorouracil and either doxorubicin or epirubicin. *J. Clin. Oncol.*, **6**, 679.

HAYWARD, J.L., RUBENS, R.D., CARCONNE, P.P., HEUSON, J.C., KUMAOKA, S. & SEGALOFF, A. (1977). Assessment of response to therapy in advanced breast cancer. *Br. J. Cancer*, **35**, 292.

HOOGSTRATEN, B., GEORGE, S.L., SAMAL, B. & 5 others (1976). Combination chemotherapy and adriamycin in patiens with advanced breast cancer. *Cancer*, **38**, 13.

JONES, R.B., HOLLAND, J.F., BHARDWAJ, S., NORTON, L., WILFINGER, C. & STRASHUN, A. (1987). A phase I-II study of intensive dose adriamycin for advanced breast cancer. *J. Clin. Oncol.*, **5**, 172.

KAPLAN, E.L. & MEIER, P. (1958). Non-parametric estimation from incomplete observations. *J. Am. Stat. Assoc.*, **53**, 451.

MOURIDSEN, H.T., ROSE, C., NOOY, M.A. & VAN OOSTEROM, A.T. (1983). Mitoxantrone as first-line cytotoxic therapy in advanced breast cancer: preliminary results of a phase II study. *Cancer Treat. Rev.*, **10** (Suppl B): 47.

NEIDHART, J.A., GOCHNOUR, D., ROACH, R.W., STEINBERG, J.A. & YOUNG, D. (1983). Mitozantrone versus doxorubicin in advanced breast cancer: a randomised crossover trial. *Cancer Treat. Rev.*, **10** (Suppl B): 41.

PEREZ, D.J., POWLES, T.J., GAZET, J.C., FORD, H.T. & COOMBES, R.C. (1984). Mitomycin C, melphalan and methotrexate, combination chemotherapy for palliation of disseminated breast cancer. *Cancer Chemother. Pharmacol.*, **13**, 36.

PETO, R., MIKE, M.C., ARMITAGE, P. & 7 others (1977). Design and analysis of randomised clinical trials requiring prolonged observation of each patient. Part 2. Analysis and examines. *Br. J. Cancer*, **35**, 1.

POWLES, T.J., SMITH, I.E., FORD, H.T., COOMBES, R.C., JONES, J.M. & GAZET, J.C. (1980). Failure of chemotherapy to prolong survival in a group of patients with metastatic breast cancer. *Lancet*, **i**, 580.

POWLES, T.J., GALLAGHER, C.J., ASHLEY, S.E., FENLON, D. & O'KEEFE, A. (1987). Mitomycin C used in combination with mitoxantrone and methotrexate as first-line cytotoxic treatment of disseminated breast cancer. In *New trends in cancer chemotherapy with mitomycin C*. Taguchi, T. & Audrysek, O. (eds). Tokyo, Excerpta Medica, pp. 51–57.

RAINEY, J.M., JONES, S.E. & SALMON, S.E. (1979). Combination chemotherapy for advanced breast cancer utilising vincristine, adriamycin and cyclophosphamide (VAC). *Cancer*, **43**, 66.

ROSNER, D., NEMOTO, T. & LANE, W. (1987). A randomised study of intensive versus moderate chemotherapy programs in metastatic breast cancer. *Cancer*, **59**, 874.

TANNOCK, I.F., BOYD, N.F., DEBOER, G. & 6 others (1988). A randomised trial of two dose levels of cyclophosphamide, methotrexate and fluorouracil chemotherapy for patients with metastatic breast cancer. *J. Clin. Oncol.*, **6**, 1377.